# Understanding clinical and non-clinical decisions under uncertainty: a scenario-based survey

**DOI:** 10.1186/s12911-016-0391-3

**Published:** 2016-12-01

**Authors:** The Writing Group for CERTAIN-CHOICES, Vlad V. Simianu, Margaret A. Grounds, Susan L. Joslyn, Jared E. LeClerc, Anne P. Ehlers, Nidhi Agrawal, Rafael Alfonso-Cristancho, Abraham D. Flaxman, David R. Flum

**Affiliations:** 1 Department of Surgery, University of Washington, Seattle, WA USA; 2 Department of Psychology, University of Washington, Seattle, WA USA; 3 Foster School of Business, University of Washington, Seattle, WA USA; 4 Surgical Outcomes Research Center (SORCE), University of Washington Medical Center, Box 354808, 1107 NE 45th St., Suite 502, Seattle, WA 98105 USA; 5 Institute for Health Metrics and Evaluation, University of Washington, Seattle, WA USA

**Keywords:** Decision making, Uncertainty, Probability, Defensive medicine

## Abstract

**Background:**

Prospect theory suggests that when faced with an uncertain outcome, people display loss aversion by preferring to risk a greater loss rather than incurring certain, lesser cost. Providing probability information improves decision making towards the economically optimal choice in these situations. Clinicians frequently make decisions when the outcome is uncertain, and loss aversion may influence choices. This study explores the extent to which prospect theory, loss aversion, and probability information in a non-clinical domain explains clinical decision making under uncertainty.

**Methods:**

Four hundred sixty two participants (*n =* 117 non-medical undergraduates, *n =* 113 medical students, *n =* 117 resident trainees, and *n =* 115 medical/surgical faculty) completed a three-part online task. First, participants completed an iced-road salting task using temperature forecasts with or without explicit probability information. Second, participants chose between less or more risk-averse (“defensive medicine”) decisions in standardized scenarios. Last, participants chose between recommending therapy with certain outcomes or risking additional years gained or lost.

**Results:**

In the road salting task, the mean expected value for decisions made by clinicians was better than for non-clinicians(−$1,022 vs -$1,061; <0.001). Probability information improved decision making for all participants, but non-clinicians improved more (mean improvement of $64 versus $33; *p =* 0.027). Mean defensive decisions decreased across training level (medical students 2.1 ± 0.9, residents 1.6 ± 0.8, faculty1.6 ± 1.1; p-trend < 0.001) and prospect-theory-concordant decisions increased (25.4%, 33.9%, and 40.7%;p-trend = 0.016). There was no relationship identified between road salting choices with defensive medicine and prospect-theory-concordant decisions.

**Conclusions:**

All participants made more economically-rational decisions when provided explicit probability information in a non-clinical domain. However, choices in the non-clinical domain were not related to prospect-theory concordant decision making and risk aversion tendencies in the clinical domain. Recognizing this discordance may be important when applying prospect theory to interventions aimed at improving clinical care.

**Electronic supplementary material:**

The online version of this article (doi:10.1186/s12911-016-0391-3) contains supplementary material, which is available to authorized users.

## Background

Clinicians routinely make decisions for and with their patients that are complex, under time constraints, and involve risks or uncertain outcomes [1, 2]. These decisions are informed by medical literature, clinical decision rules, predictive models, and, in practice, a number of subjective “rules of thumb” or heuristics that may deviate from what is considered economically “rational.” Decision scientists, psychologists, and behavioral economists have identified many such “decisional short-cuts” that impact judgment and decision making [3, 4], and some have extended heuristic-based decision theories to clinician decision making. While empiric evidence for the application of these decision theories to medical decision making is increasing [5–7], it remains to be determined to what extent heuristic-based decision theory, developed and validated in non-medical realms, can be validated in real-world, health-related decision making.

For instance, prospect theory holds that people have a tendency to make risk-seeking decisions in cost/loss situations. For example, they would rather risk a greater cost than incur a certain, lesser cost to protect themselves against the greater loss [8, 9]. This pattern of behavior, an important part of prospect theory, asserts that individuals make decisions based on the gains or losses associated with possible outcomes, and that losing something causes more mental distress than gaining something of the same value [10]. However, the tendency toward taking such risks may differ from one person to another [11], due to level of expertise with uncertainty information [12–14], or may differ from one domain (e.g. health or finance) to another [15–17]. Furthermore, research suggests that people make better decisions when provided with explicit numeric uncertainty information [18, 19]. Numerical uncertainty is commonly expressed as probability, and probability estimates are increasingly used to quantify risk in both nonmedical and medical domains [18, 19]. However, prospect theory was developed in non-clinical domains with non-clinical participants, and there are gaps in the empirical evidence of the utility of these theories to explain clinical decision making of doctors.

To explore the applications of prospect theory, this study aims to build on growing medical decision making research activities over the last several decades by describing some unique decision making in scenarios with uncertainty by doctors. To assess prospect theory in these scenarios, we compared clinicians’ decision making with that of non-clinicians, in non-clinical and clinical domains by assessing to what extent expertise and level of training impact decisions with uncertainty and risk-preference. Specifically, we hypothesized that doctors (perhaps due to advanced education or higher numeracy [18, 19]) are better able to take advantage of explicit probability than are non-doctors, and make more economically-rationale decisions. Our second hypothesis was that the risk propensity of doctors (in other words, prospect-theory concordance in their decisions) is stable across medical and non-medical domains and that risk aversion in non-medical tasks would reflect risk aversion in medical tasks. Our third hypothesis was that better use of probability information would be associated with less risk-inclined decision making (increasing prospect-theory concordance when probability information is provided). To test these hypotheses, subjects performed a three-part, web-based study that tested 1) non-medical decision making with and without explicit probability information; and described medical decision risk preference using 2) defensive medicine scenarios and 3) gain/loss scenarios.

## Methods

This study was approved by the University of Washington Institutional Review Board.

### Study population

Undergraduate students enrolled in the introductory Psychology course at the University of Washington and all medical students, residents, and faculty physicians at the University of Washington were eligible. Potential participants were contacted via email and were provided a personalized link to the three online tasks described below. Participants were encouraged to complete the three tasks in one computer session at the participants’ discretion/choice of location. All participants were reimbursed $5 for participating, and up to $10 based on performance in the decision task described below. Results of 11 participants who began the tasks but failed to complete them were excluded from analysis. In all, 462 participants’ results were included for analysis (*n =* 117 undergraduates, *n =* 113 medical students, *n =* 117 residents, *n =* 115 faculty physicians).

After providing informed consent, participants reported their demographic characteristics and completed three sequential tasks, as described below: a simulated road salt decision task, a defensive medicine task and a medical risk preference task. While many participants self-identified as subspecialists, for descriptive purposes their specialties were categorized as Surgical (Surgery, Orthopaedics, Neurosurgery, Urology, etc.), Medical (including Emergency Medicine), Pediatric (including Adolescent Medicine) or Other (including Anesthesia, Radiology, Pathology). Undifferentiated medical students were asked to report medical specialty of interest.

### Road salt decision task

In this task, modified to be completed in an online format from Joslyn and LeClerc [20], participants assumed the role of president of a road maintenance company, in contract with a U.S. town to treat its roads for a two-month period in winter to prevent icing. Over the course of 60 trials, participants decided whether, based on weather forecasts for nighttime low temperatures, treating the roads with salt brine was warranted. They received a virtual monthly budget of $36,000 for each of the two hypothetical months of the game. Applying salt brine cost $1,000 per day. Not applying salt brine cost nothing, but if a freezing temperature occurred, participants were penalized $6,000. Thus, the decision task represented a situation in which one had to decide between paying to protect oneself against a potential loss and taking the risk that freezing temperatures would not occur that night. Prospect theory suggests that people tend to be risk seeking in such situations [8, 9].

Participants were instructed to maximize profits by minimizing salting expenses and avoiding penalties. They were told that they would receive a cash reward at the end of the experiment commensurate with their ending balance ($1 for every $1000 above $12,000, the amount that would remain if they choose to salt every day). In each trial, representing one day, a forecast for the next night appeared on the screen. Participants indicated their decision to apply salt brine to the roadways or not by clicking on one of two boxes marked “Salt” or “Not salt.” Immediately afterward, the actual nighttime low temperature and any balance adjustments appeared on the screen. Participants were able to continue even if their balance dropped below $0 by borrowing against the next month’s installment. After a break screen appeared at the end of the first month, participants clicked “Next” to continue on to the next month’s trials, and the budget was increased by $36,000.

Forecast format was randomized between groups. There were two conditions: a control condition and a probability condition. In both conditions, participants saw a forecast, e.g.: “The expected nighttime low temperature for tomorrow is 35 °F”. In the control condition, this was the only information in the forecast. In the probability condition, participants were told the percent chance that temperatures would be at or below freezing: “The expected nighttime low temperature for tomorrow is 35 °F and there is a 22% chance that the temperature will be equal to or less than 32 °F”(Additional file 1). At the end of the second month, participants were informed of their ending balance and amount of cash reward.

There were two main outcomes reported for this portion of the study, intented to characterize the influence of prospect theory on decision making: expected value and percent salting decisions above and below threshold. Overall quality of decisions was reflected in expected value, calculated for each participant for each trial day (60 total). Expected value was the penalty (−$6,000) multiplied by the probability of freezing for each trial on which participants decided not to salt. Participants who decided to salt were assigned the cost of salting (−$1,000). A mean expected value score for all trials was calculated for each participant. The expected value measure is a surrogate for overall quality of decisions made in the salting task, but does not account for where salting decision systematically deviated from the optimal decision. To that end, we also report percent of decisions to salt below and above the economically optimal threshold of 17% probability of freezing ($1000 divided by $6000). When the probability of freezing is below 17%, the optimal decision is *not* to salt as salting would be expected to cost more than the probability of freezing multiplied by the penalty of $6000. Salting below 17% would therefore be considered a risk-averse decision. When the probability of freezing is above 17%, the optimal decision is to salt as salting would be expected to cost less than the probability of freezing multiplied by the penalty of $6000. Not salting above 17% probability of freezing would be considered risk-seeking. Our hypothesis was that medical participants completing these scenarios would have a higher expected value of decisions, and would improve more than non-medical participants when provided probability information.

### Defensive medicine

Subsequent to completion of the salting trials, participants were asked to answer questions about a series of four medical scenarios (Additional file 2), adapted with permission from Klingman et al. [21] These questions, drawn from clinical situations used in board certification examinations, were developed to empirically characterize the extent to which defensive medicine affects clinical practice [21]. In all of the scenarios, participants were given the choice between (A) ordering a defensive medical test or procedure and (B) declining further management, an option medically acceptable by the expert panels. These scenarios depict defensive medicine practice as “doing something” where not pursuing further care would be acceptable. Prospect theory would suggest that selecting the “doing something” decision follows the principle of loss aversion (less risky than doing nothing). Acknowledging that the practice of defensive medicine varies considerably across clinical situations (in other words, a clinician may be defensive in one situation but not in another) [22], the main outcome reported for each participant was total “defensive score,” a sum of defensive decisions made across the four scenarios. Our hypothesis was that medical participants completing these scenarios would make fewer “defensive” decisions as their level of training (a surrogate for experience) increased. We hypothesized also that risk-averse (or defensive) decisions in these scenarios would be associated with risk-averse decisions in the other two tasks.

### Nightingale risk preference instrument

Following the defensive medicine scenarios, participants read two scenarios developed by Nightingale (Additional file 3) designed to ascertain willingness to gamble on behalf of patients in the face of gain or loss [23–25]. In the first scenario focusing on gains, selecting **option A** represented a preference for a moderate gain and no chance of failure. Selecting **option B** represents a preference for a chance for significant gain, but also a risk of complete failure. The second scenario was similar, but evaluated willingness to accept loss for the patient: **option A** minimized loss whereas **option B** subjected the patient to a smaller risk of great loss and a possible risk of no loss. According to prospect theory [8, 9], people prefer a certain gain, but avoid a certain loss (in other words, they should select answer A in the first case, and B in the second). To the extent that this is true, this instrument has been proposed to evaluate the degree to which a physician is considered risk seeking or risk averse. Those who refuse to gamble in the face of loss are considered risk averse [26].

Responses for participants were organized into four categories: (1) chose the certain option in both cases (risk averse as above), (2) chose the risky option in both cases, (3) prospect theory concordant (gambled on loss scenario only), and (4) prospect theory discordant (gambled on gain scenario only). We hypothesized that as level of medical training increased, participants would make fewer risk-averse decisions.

### Statistical analysis

Patient characteristics and outcomes were summarized using frequency distributions for categorical variables, and means and confidence intervals (95% CI) for continuous variables. We stratified our description by medical versus non-medical status, as well as levels of self-identified medical training (medical student, resident, faculty). Categorical variables were compared using the Pearson χ2 statistic. To avoid multiple comparisons, continuous variables were compared using an Analysis of Variance (ANOVA) with post hoc pairwise comparisons [20]. For the salting task, mean expected value is reported stratified by medical versus non-medical participants and presentation of explicit probability information. In addition, mean salting decisions below and above the 17% threshold probability for freezing are reported, stratified by medical versus non-medical participants and by availability of explicit probability information. Linear regression models were used to evaluate the association of increasing age with outcomes in the salting task after adjustment for level of medical training and probability type.

For the defensive medicine task, mean defensive score and standard deviation (SD) by participant level is reported. For the nightingale risk preference instrument, frequency distributions based on participant level are reported. Trends are reported using linear regression models for a particular decision using level of training as a continuous variable. Adjustment was done for medical specialty, modeled as a categorical variable. Comparisons were also made across the three tasks using ANOVA models as above. Specifically, we report the correlation decisions in the iced roads task (expected value and overall salting choices) with defensive medicine score as well as with risk aversion and prospect-concordant decision-making in the Nightingale instrument. All correlation models controlled for education level of participants. A *p*-value of less than 0.05 was considered statistically significant. All analysis was performed using SPSS version 19 (Armonk, NY: IBM Corp.).

## Results

The demographics of the 462 participants (age range 18–74, 47% male) are shown in Table 1. Each level represented approximately 25% of the participant population. There were expected age and gender gradients across levels of medical training.

**Table 1:** Demographics of Non-Medical and Medical study participants

	Non-Medical		Medical Students		Residents		Faculty		Total	
Variable	*N =* 117	25%	*N =* 113	24%	*N =* 117	25%	*N =* 115	25%	*N =* 462	100%^a^
Median age [range], years	20	[18–27]	25	[21–36]	30	[25–36]	49	[30–74]	28	[18–74]
Male	50	43%	41	37%	56	48%	69	61%	216	47%
Medical Specialty^b^										
Medicine	-	-	63	56%	29	25%	40	35%	132	38^%c^
Surgery	-	-	25	22%	56	48%	37	32%	118	34%^c^
Pediatrics	-	-	15	13%	27	23%	26	23%	68	20^%c^
Other/undecided	-	-	10	9%	5	4%	12	10%	27	8^%c^

^a^Total may not add up to 100% due to rounding

^b^Are listed in descending order of largest proportion of total study cohort

^c^Out of total of medical participants, n = 345

### Salting task

**Fig. 1:**
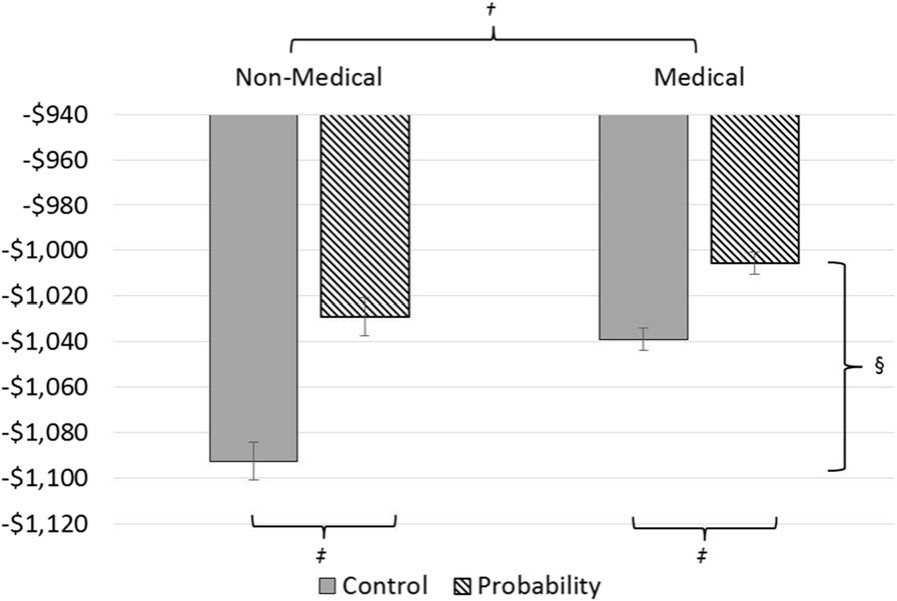
Expected Value estimates of Non-medical and Medical participants, stratified by presentation of explicit probability information. *Medical participants include medical students, residents, and faculty physicians. †Medical participants made better decisions than non-medical participants (mean expected value -$1022 versus -$1,061; *p* < 0.001). ‡Participants receiving probability information made better decisions than those presented with control control scenarios (mean expected value -$1017 versus -$1066; *p* < 0.001), but probability^§^ helped non-medical participants improve more than medical participants (mean improvement of $64 versus $33; *p* = 0.027)

**Fig. 2:**
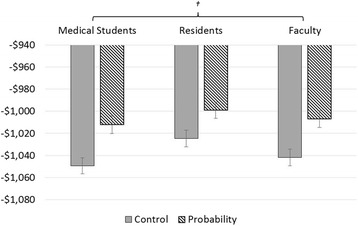
Mean expected values of across training level of medical participants, stratified by presentation of explicit probability information. *Participants receiving probability information made better decisions than those receiving control scenarios (*p* < 0.001), but this † difference did not change with medical education level (*p* = 0.72)

Overall, 48.9% of participants (226 of 462) received explicit probability information in this task: n_non-medical_ =59 (50.4%); n_medical students_ = 50 (44.2%); n_residents_ = 59 (50.4%); n_faculty_ = 58 (50.4%). The mean expected value for medical participants (−$1022; 95% CI: −$1029, −$1016) was better than for non-medical participants (−$1061; 95% CI: −$1073, −$1049; *p <* 0.001) (Fig. 1). This suggests that medical participants made overall better decisions in this task than non-medical participants. Mean expected value for participants receiving probability forecasts (−$1017; 95% CI: −$1027, −$1008) was higher than for those in the control condition (−$1066; 95% CI: −$1075, −$1056; *p <* 0.001). Moreover, probability information helped non-medical participants more than medical participants (mean improvement of $64 versus $33; *p =* 0.027). Age differences across the two groups did not contribute any additional explanation for differences in quality of decisions (*p =* 0.15). The improved expected value with explicit probability information persisted across the three levels of medical training (*p <* 0.001) (Fig. 2). However, this improvement did not differ across levels (*p =* 0.72). Age differences across the three groups did not contribute any additional explanation for differences in quality of decisions (*p =* 0.80).

**Fig. 3:**
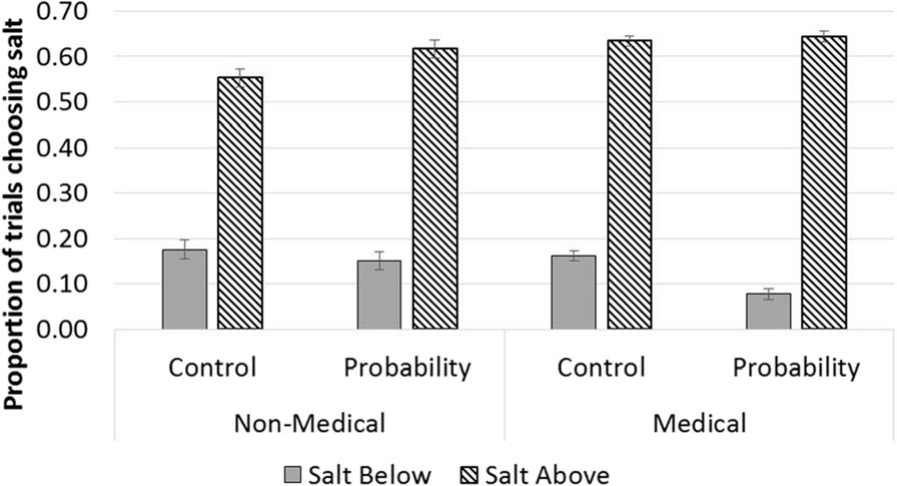
Overall salting decisions of Non-medical and Medical participants, stratified by presentation of explicit probability information. *Medical participants salted less below 17% and more above, *p* < 0.001. Participants receiving probability salt less below 17% and more above, *p* < 0.001. Despite the appearance that probability information may have helped non-medical and medical participants differently, there was no evidence of interaction between medical status and probability information condition (*p* = 0.87)

Medical participants salted less below the 17% threshold (mean 12.0%; 95% CI: 10.4%, 13.6%; versus 16.4%; 95% CI: 13.6%, 19.2%) (Fig. 3), and more above (mean 63.9%, 95% CI: 62.3%, 65.5% versus mean 58.4%, 95% CI: 55.6%, 61.2%) than did non-medical participants (*p <* 0.001). As compared to participants using control condition forecasts, participants receiving probability forecasts salted less below 17% (mean 11.5%; 95% CI: 9.2%, 13.7%; versus mean 16.9%; 95% CI: 14.6%, 19.2%) and more above (mean 63.0%; 95% CI: 60.7%, 65.3%; versus mean 59.3%; 95% CI:57.0%, 61.6%; *p <* 0.001). While receipt of explicit probability information appeared to help medical participants by decreasing decisions to salt below the threshold and non-medical participants by increasing decisions to salting above the threshold, this trend did not reach statistical significance (*p =* 0.87). Age of participant did not have any additional effect on salting below or above the threshold (*p =* 0.24 and *p =* 0.18, respectively).

### Defensive medicine

**Fig. 4:**
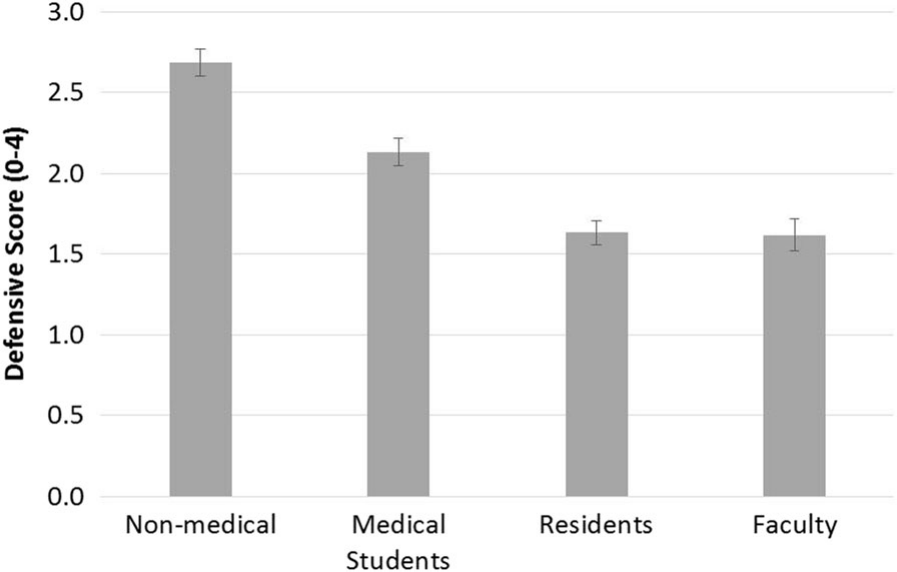
Mean defensive medicine score by training level of participants. *Increasing medical education level results in less defensive medicine (p-trend <0.001)

Mean total defensive medicine scores decreased across training level of participants (p-trend <0.001) (Fig. 4). Undergraduate participants had the highest mean defensive score of 2.7 ± 0.9 (out of 4). Interestingly, the trend in decreasing defensive scores with levels of training persisted in the subgroup of medical participants after adjustment for medical specialty, with medical students having mean defensive scores of 2.1 ± 0.9, residents having a mean of 1.6 ± 0.8 and faculty having a mean of 1.6 ± 1.1 (p-trend < 0.001).

### Nightingale risk assessment

Responses to the Nightingale risk assessment instrument were categorized as those who never took a gamble, those who always took a gamble, those who took a gamble to avoid a loss (prospect theory concordant) and those who took a risk in the face of a gain only (inconsistent with prospect theory) (Table 2). Of all groups of participants, undergraduates had the highest proportion that always chose to gamble 30.8%. The majority of medical participants (*n =* 345) either never took a gamble (*n =* 136, 39%) or only took a gamble to avoid a loss (*n =* 118, 34%). Among medical participants, the proportion who never chose to gamble (in other words, who were risk-averse) decreased with increasing medical education (48.7% of medical students, 39.3% of residents, and 30.4% of faculty p-trend = 0.005). In addition, the proportion of medical participants who made prospect theory-concordant decisions (in other words, chose to only gamble to avoid a loss) increased with medical education level (26.5% of medical students, 34.2% of residents, and 41.7% of faculty, p-trend = 0.016). There was no evidence of effect of medical specialty across Nightingale gamble choice category (all *p >* 0.1).

**Table 2:** Nightingale risk category by participant level (all participants, *n =* 462)

	Never gamble		Always gamble		Prospect theory concordant		Prospect theory discordant	
	*N =* 171	37.0%*	*N =* 80	17.3%*	*N =* 149	32.3%*	*N =* 62	13.4%*
Non-medical^a^	35	29.9%	36	30.8%	31	26.5%	15	12.8%
Medical Students^b^	55	48.7%	14	12.4%	30	26.6%	14	12.4%
Residents^c^	46	39.3%	14	12.0%	40	34.2%	17	14.5%
Faculty^d^	35	30.4%	16	13.9%	48	41.7%	16	13.9%

* Reported as percentage of all participants (n = 462)

^a^Reported as percentage of all non-medical participants (n = 117)

^b^Reported as percentage of all medical students (n = 113)

^c^Reported as percentage of all residents (n = 117)

^d^Reported as percentage of all faculty (n = 115)

### Risk aversion across tasks

Beyond differences in decision making between medical and non-medical participants and their use of probability information, we found no relationship between decision making in the salting scenarios and medical scenarios. There was no evidence of correlation of defensive score to expected value (*p =* 0.07) or to salting decisions (*p =* 0.97). In addition, there was no correlation between risk-aversion in the Nightingale instrument (never gambling) and expected value (*p =* 0.16) or salting decisions (*p =* 0.14). There was no correlation between prospect theory concordant decisions (gambling to avoid a loss only) and expected value (*p =* 0.26) and salting decisions (*p =* 0.94).

## Discussion

We found that in a non-medical domain, both clinicians and non-clinicians made more economically rational decisions when given explicit probability information. Clinicians showed less improvement with probabilities than did non-clinicians. However, baseline performance was better for clinicians, which might suggest a ceiling effect to improvement. Additionally, probability information may have helped clinicians differently, by allowing them to salt less below the economically optimal threshold (suggesting fewer risk-averse decisions). For undergraduates, presentation of probability information increased salting above the threshold. No differences in decision making in the non-medical task were noted across levels of medical training for clinicians. Choices in the non-medical domain were not related to prospect-theory-concordant decision making and risk aversion tendencies in the medical domains that were tested.

This study explored whether clinician decision making is different than decision making by non-clinicians. Because non-clinicians don’t have experience with making medical decisions, we chose a task in which everyone has equal content expertise (weather and road-salting task) [20], although clinicians might be expected to make better use of probability information in this task because of advanced education and/or numeracy [18, 19]. Our findings indicate that in this task, clinicians *did* make better quality decisions overall. There are several possible explanations for this. It may be due to additional training in making decisions in situations with uncertainty or perhaps due to self-selection into the medical field. However, this difference was not age-dependent. These findings support the assertion that providing explicit probability information improves decisions [18, 19]. However, it would appear clinicians have a different baseline and explicit probability information may help ‘novices’ more.

We found that both defensiveness and risk aversion decreased with increasing medical experience. We measured risk aversion three different ways in this study. In the first, non-medical task, risk-averse errors would involve choosing to salt when the probability of freezing was below the economically optimal threshold of 17% (Fig. 3). To that end, it would appear qualitatively that explicit probability information reduced risk aversion more in clinicians. However, when compared to non-clinical undergraduates this reduction did not reach significance, perhaps because risk averse-errors were rare, overall. In the defensive medicine scenarios, risk aversion manifested as a higher total of “defensive decisions.” In the Nightingale instrument, risk aversion was defined by lack of gambling for either years gained or years lost. In the latter two tasks we saw that risk aversion decreased with increasing medical experience. However, the lack of correlation between these medical measures of risk aversion and risk aversion in the salting task challenges the notion that risk aversion crosses domains. Indeed, research dating back to the 1960s argued financial risk taking might not be a good predictor of other risk taking areas [27]. To that end, the disconnect between domains found in our study supports the assertion that medical decision making is different for clinicians, and these findings raise awareness of studying risk preference in a domain-specific way [15–17].

One possible explanation for the differences in decision patterns and risk preference across domains may come from the affect involved in healthcare-related decisions [28]. Several behavioral studies have identified differences in decision making between affect-rich and affect-poor tasks [29–33]. This distinction is important because multiple models of decision making under risk share the common notion that outcomes are weighted by their probability, maximizing the expected outcome. However, when faced with emotion or affect-charged decisions, evidence suggests that people systematically choose the optimal option less often [28]. And others have suggested that the impact of probability information may be attenuated in affect-rich choices [34]. Though this was not explicitly studied in this set of tasks, the affect involved in choices made by physicians in health-care decisions may be different than that of non-clinicians, and may impact the measured risk preferences in these situations.

Another distinction between clinical and non-clinical decisions involves the “agent effect,” in which making decisions for others may invoke different levels of loss aversion [35, 36]. While most decision making theory has focused on situations where subjects choose for themselves, healthcare poses a unique environment where clinicians are often delegated to make choices for others. In financial domains, it has been shown that loss of others’ money would not trigger an equal amount of emotional distress as loss of one’s own money [37, 38]. Furthermore, it is unclear how risk preferences change when shifting from self to others, with evidence existing to support a change to more risk-averse [39], no difference [40], or more risk-seeking decisions [41–43]. With this in mind, the disconnect between risk aversion in the first, non-medical task, and the second and third, medical-domain tasks may come from a shift in decision making for self (salting) versus others (patients in medical scenarios). In that regard, the findings in this study highlight a potentially significant challenge to the translation of classic decision theories or behavioral economics to decision architecture aimed at improving clinician decision making.

These findings should be considered in light of several limitations. First, this is the first application of these tasks using an online questionnaire. For feasibility reasons, it was not possible to administer this task in a standard laboratory across this spectrum of medical personnel. Furthermore, our study is subject to selection bias towards those participants who chose to participate through email recruitment. While it is reassuring the findings of the salting task were similar to the findings of prior evaluations of the same task in the laboratory setting, there may be more variation in subjects’ attention, time, and investment in completing the task in an online format. Since this is the first online application of both the defensive scenarios [21] and the Nightingale instrument [23–25], it is unclear how these responses may differ. Indeed, there is evidence to suggest that subjects’ decision-making in the controlled, laboratory setting, may be different in the ‘real world’ where the real impact of penalties incurred by their decisions is more tangible [44]. Second, our study may have been underpowered to detect important differences in task responses. Group size was determined using estimates (previously established by Joslyn and LeClerc [20]) of the sample size needed to detect a difference in decision quality between control and probability scenarios. To that end, we may have been underpowered to detect differences between risk aversion and risk seeking behavior and differences across domains or certain demographic variables like age, gender, and medical specialty. For instance, age is typically associated with reduce risk taking. This was not borne out in our results, but may be because categories of medical training already explained much of this variation, and our study was not powered to detect differences in age, gender, or medical specialty. Moreover, the tasks differ in important ways. For example, the “road salt” task involves repeated decisions with feedback. There is some research to suggest that behavior in these types of decisions deviates drastically from those made in descriptive contexts like in the defensive medicine scenarios [45, 46]. In addition, our defensive medicine scenarios don’t suggest the costs of the defensive choice, which faculty may know and medical students may not. Thus, the differences may not reflect differences in loss aversion or risk attitude, but in knowledge. Finally, we categorized expertise two ways – clinician and non-clinician, and across levels of medical education (student, resident, faculty). In reality, expertise (in both numeracy and medical knowledge) happens across a continuum and is not strictly based on the categorization of one’s position.

## Conclusions

These limitations non withstanding, this study adds valuable insight into risk preferences across clinical and non-clinical simulations in doctors, and more specifically across levels of medical training. Medical training is expected to change one’s experience with making decisions under uncertainty and accordingly, we found trends in risk aversion and prospect theory concordance across education levels. In addition, the findings of this study demonstrate that clinicians performed as predicted in a non-clinical decision making experiment, with small differences perhaps explained by advanced numeracy. However, a lack or association between decision patterns made across clinical and non-clinical scenarios—that fact the so-called financial decisions do not predict medical ones—challenges the application of classic decision theories to those made by clinicians, either because of the affective aspect of clinical decisions or because of their role as agents. Understanding which aspects of decision science translate into the clinical realm and how clinicians may or may not behave differently than non-clinicians is important because it may support the crafting of interventions to improve clinicians’ decisions in situations of uncertainty.

## Additional files

Additional file 1: Sample salting questions as portrayed on the screen of the individual participants: control (1A) and probabilistic (1B) versions. Red arrow depicts addition of explicit probability information for probability scenarios (1B). Actual temperature was displayed each day, after decision to salt or not had been made (1C). (ZIP 71 kb)
Additional file 2: Questions about a series of four medical scenarios (DOCX 14 kb)
Additional file 3: Nightingale’s two-question format (DOCX 13 kb)
