# Supplementary figures and images for: Understanding clinical and non-clinical decisions under uncertainty: a scenario-based survey
Source: BMC Med Inform Decis Mak. 2016 Dec 1;16:153. doi: 10.1186/s12911-016-0391-3 (PMC5131551; doi:10.1186/s12911-016-0391-3)

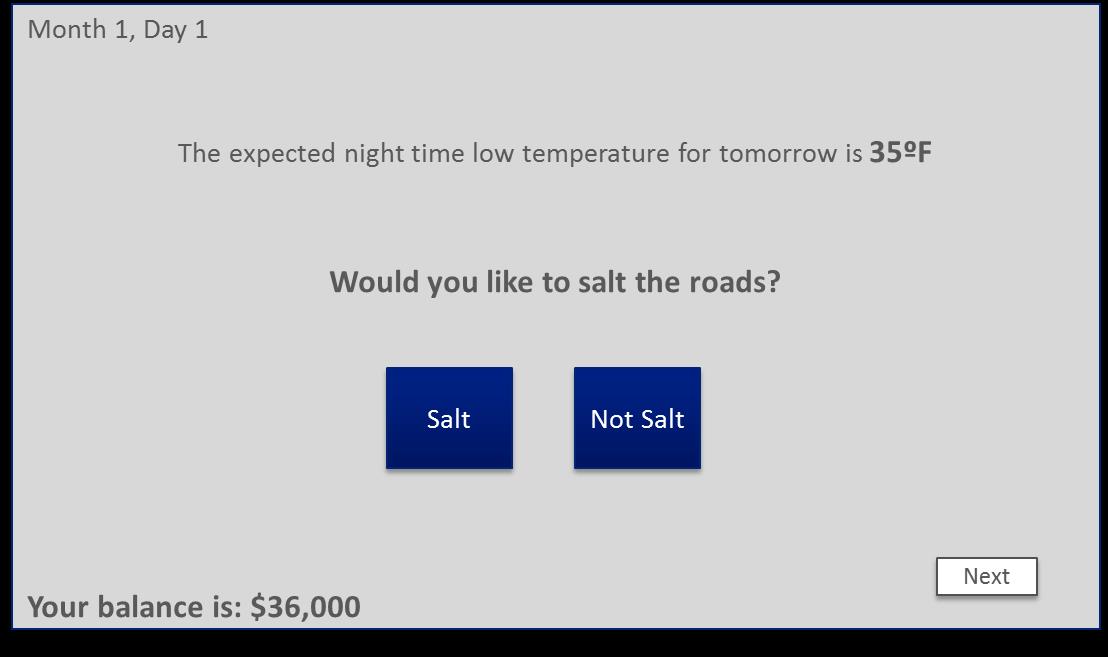

Supplement: Additional file 1: — Sample salting questions as portrayed on the screen of the individual participants: control (1A) and probabilistic (1B) versions. Red arrow depicts addition of explicit probability information for probability scenarios (1B). Actual temperature was displayed each day, after decision to salt or not had been made (1C). (ZIP 71 kb) [file 12911_2016_391_MOESM1_ESM.zip › Additional file 1/Appendix 1A.pngR2.jpg]

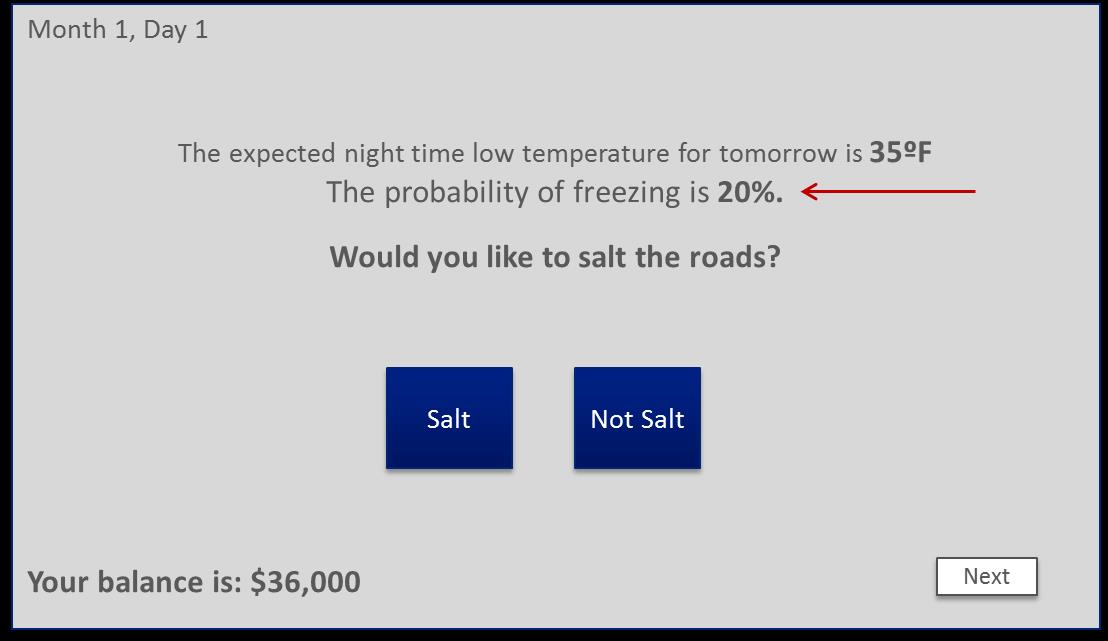

Supplement: Additional file 1: — Sample salting questions as portrayed on the screen of the individual participants: control (1A) and probabilistic (1B) versions. Red arrow depicts addition of explicit probability information for probability scenarios (1B). Actual temperature was displayed each day, after decision to salt or not had been made (1C). (ZIP 71 kb) [file 12911_2016_391_MOESM1_ESM.zip › Additional file 1/Appendix 1BR2.jpg]

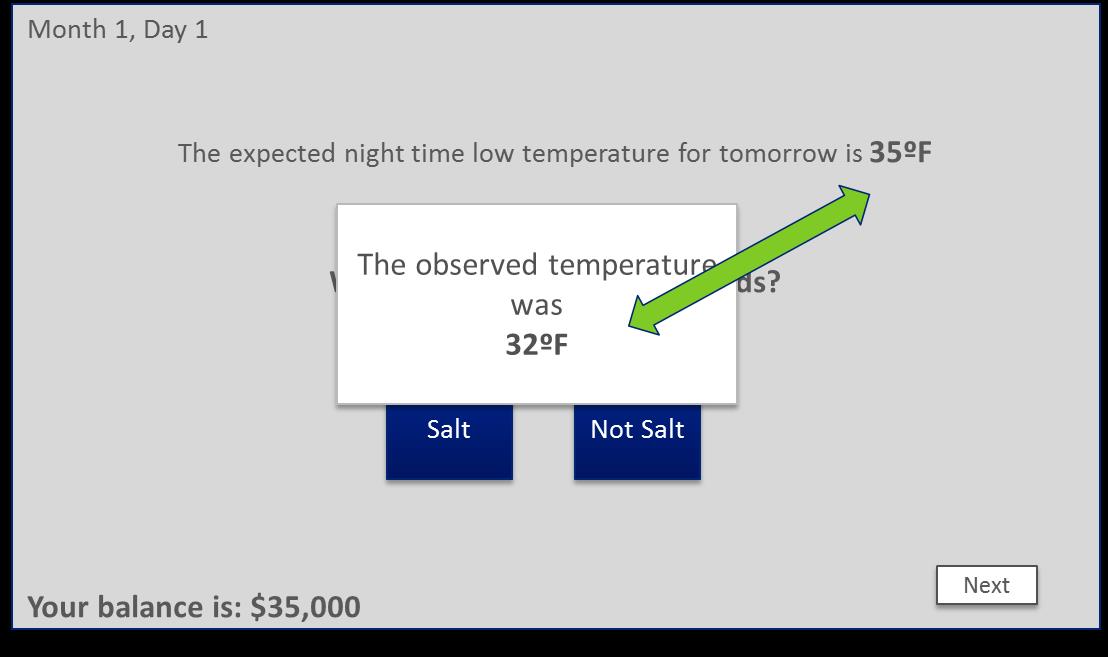

Supplement: Additional file 1: — Sample salting questions as portrayed on the screen of the individual participants: control (1A) and probabilistic (1B) versions. Red arrow depicts addition of explicit probability information for probability scenarios (1B). Actual temperature was displayed each day, after decision to salt or not had been made (1C). (ZIP 71 kb) [file 12911_2016_391_MOESM1_ESM.zip › Additional file 1/Appendix 1CR2.jpg]
