# Supplementary material for: Understanding clinical and non-clinical decisions under uncertainty: a scenario-based survey
Source: BMC Med Inform Decis Mak. 2016 Dec 1;16:153. doi: 10.1186/s12911-016-0391-3 (PMC5131551; doi:10.1186/s12911-016-0391-3)
Supplement: Additional file 2: — Questions about a series of four medical scenarios (DOCX 14 kb) [file 12911_2016_391_MOESM2_ESM.docx]

**Additional file 2:** Participants were asked to make decisions in the following “Defensive medicine” scenarios. *Adapted with permission from Klingman et al.* ^17^

**Scenario 1**

**History of Present Illness**: A 42-year-old man arrives at the emergency room complaining of chest pain. The pain is on the left side and is worse when he changes position. While it is sore to the touch, he states that it feels “deep.” The pain has persisted for 1 hour. He has not experienced chest pain previously. He jogs 3 times a week and does not smoke. He had a normal routine physical examination a week ago.

**Physical Examination:** The patient is tense and anxious. His blood pressure is 140/80; his heart rate is 80. The anterior chest wall is tender over the left sternal border. Examination of the heart and lung is normal.

**Additional Data**: A 12-lead electrocardiogram and chest X ray are normal. Laboratory tests, including a complete blood count, electrolytes, and cardiac enzymes, are normal.

Please select one of the following:
A. Admit for observation / additional testing
B. Discharge the patient

**Scenario 2**

**History of Present Illness**: A 35-year-old man comes to your office complaining of bright red blood per rectum. Over the past 4 days he has observed a few drops of blood in the toilet and on the toilet paper after having a bowel movement. He denies any recent change in bowel habits and has otherwise been in good health.

**Physical Examination:** Rectal examination is normal.

**Additional Data**: Anoscopy reveals nonbleeding internal hemorrhoids. A hemoccult is positive. A hemoglobin, hematocrit, carcinoembryonic antigen, and flexible sigmoidoscopy are all within normal limits.

Please select one of the following:
A. Order colonoscopy OR air-contrast-enema
B. Do nothing and re-examine in 2 months

**Scenario 3**

**History of Present Illness**: A 15-year-old boy fell from his skateboard after riding over a crack in the sidewalk. He hit his head, got up, and skated home. Thirty minutes after the fall he told his mother about the incident and she brought him to the ER. In the ER, the patient admits to light-headedness and some tenderness at the site of impact.

**Physical Examination:** There is an area of tenderness and swelling at the left parietal area. Mental status and neurological exam are normal.

Please select one of the following:
 A. Order CT of head
 B. Discharge under surveillance of mother

**Scenario 4**

**History of Present Illness**: A 52-year-old man is seen by you in your office. He complains of back pain and numbness of his right great toe for the past week. He attributes the injury to driving over a pothole in his pickup truck. He has been able to continue to work since the injury.

**Physical Examination:** The patient has decreased range of motion of his back. There is lumbosacral spasm. He has decreased sensitivity along the medial aspect of his right lower leg. Straight-leg raising produces right leg discomfort at 70 degrees. Ankle jerks are slightly diminished bilaterally; however, no other motor or sensory deficits are revealed on exam. There are no bowel or bladder complaints. The rest of the exam is normal.

Please select one of the following:
 A. Order imaging of the spine
 B. Recommend bedrest and follow-up in 1 week
