# Supplementary material for: Understanding clinical and non-clinical decisions under uncertainty: a scenario-based survey
Source: BMC Med Inform Decis Mak. 2016 Dec 1;16:153. doi: 10.1186/s12911-016-0391-3 (PMC5131551; doi:10.1186/s12911-016-0391-3)
Supplement: Additional file 3: — Nightingale’s two-question format (DOCX 13 kb) [file 12911_2016_391_MOESM3_ESM.docx]

**Additional file 3**: Nightingale’s two-question format tests the respondent’s willingness to gamble for their patients in both the face of gain and in the face of loss.

**Scenario**

How would you handle the following decision? Please select either A or B.
Choose between two new therapies for a **healthy** person:

A. A 100% chance of living 5 years more than the average person
A 0% chance of living 0 years more than the average person

B. A 50% chance of living 10 years more than the average person
A 50% chance of living 0 years more than the average person

**Scenario**

How would you handle the following decision? Please select either A or B.
Choose between two new therapies for a **sick** person:

A. A 100% chance of living 5 years less than the average person
A 0% chance of living 10 years less than the average person

B. A 50% chance living just as long as the average person
A 50% chance of living 10 years less than the average person
